# Urinary Concentrations of Metals and Metalloids in Malaysian Adults

**DOI:** 10.1007/s12403-021-00390-z

**Published:** 2021-07-01

**Authors:** Zurahanim Fasha Anual, Noraishah Mohammad Sham, Rashidah Ambak, Fatimah Othman, Rafiza Shaharudin

**Affiliations:** 1 Environmental Health Research Centre, Institute for Medical Research, National Institutes of Health, Ministry of Health, Shah Alam, 40170 Malaysia; 2 Centre for Nutrition Epidemiology Research, Institute for Public Health, National Institutes of Health, Ministry of Health, Shah Alam, 40170 Malaysia; 3 Dietetic and Food Service Department, Hospital Sultanah Aminah, Ministry of Health, Johor Bahru, 80000 Malaysia

**Keywords:** Biological monitoring, Heavy metals, Arsenic, Cadmium, Lead, General population

## Abstract

Exposure to environmental pollutants in humans can be conducted through direct measurement of biological media such as blood, urine or hair. Assessment studies of metals and metalloids in Malaysia is very scarce although cross-sectional nationwide human biomonitoring surveys have been established by the USA, Canada, Germany, Spain, France, and Korea. This study aims to assess urinary metal levels namely cadmium (Cd), nickel (Ni), lead (Pb) and arsenic (As) among Malaysian adults. This was a cross-sectional study involving 1440 adults between the age of 18 and 88 years old. After excluding those with 24 h urine samples of less than 500 ml, urine creatinine levels < 0.3 or > 3.0 g/L and those who refuse to participate in the study, a total of 817 respondents were included for analysis. A questionnaire with socio-demographic information such as age, gender, occupation, ethnic, academic qualification and medical history was administered to the respondents. Twenty-four-hour urine samples were collected in a container before being transported at 4 °C to the laboratory. Samples were then aliquoted into 15 ml tubes and kept at − 80 °C until further analysis. Urine was diluted ten-fold with ultrapure water, filtered and analysed for metals and metalloids using Inductively Coupled Plasma-Mass Spectrometry (ICP-MS). The geometric mean of urinary As, Ni, Cd and Pb concentrations among adults in Malaysia was 48.21, 4.37, 0.32, and 0.80 µg/L, respectively. Males showed significantly higher urinary metal concentrations compared to females for As, Cd and Pb except for Ni. Those who resided in rural areas exhibited significantly higher As, Cd and Pb urinary concentrations than those who resided in urban areas. As there are no nationwide data on urinary metals, findings from this study could be used to identify high exposure groups, thus enabling policy makers to improve public health strategically.

## Introduction

Toxic metals and metalloids pose health risk to humans due to their abundance in nature as they are widely deposited in air, soil and water. They have no role in human physiology and may cause intoxication. The most common source of exposure in the general population is through oral intake especially from water and food although others may be exposed through occupation or contact with other matrices in the environment (ATSDR 2012). Chronic exposure to metals and metalloids may potentially affect human health in long term and is a cause for concern for health agencies.

Humans may be exposed to multiple forms of arsenic (As), namely, organic and inorganic As. The more toxic form, the inorganic arsenic, is an established human carcinogen and it might also cause non-carcinogenic effects such as developmental retardation, cardiovascular and metabolic disease (IARC 2002; Nava-Acien et al. 2009). The general population are usually exposed to inorganic As from drinking water and eating contaminated food (rice and grains) (EFSA 2009), while exposure to organic As is mainly from consumption of fish and seafood (Fransesconi and Kuehnelt 2004). Cadmium (Cd) is a component of earth’s crust, naturally occurring in the environment, as a result of erosion and classified as group 1A carcinogen (ATSDR 2012). Production of nickel–cadmium batteries is one of the major source of Cd in the environment (ATSDR 2012). Other human exposures are from cigarette smoking and contaminated food (soybean, rice, leafy green, nuts and pulses) (EFSA 2009).

Human biomonitoring (HBM) can be defined as “the method for assessing human exposure to chemicals or their effects by measuring these chemicals, their metabolites or reaction products in human specimens” (CDC 2005). HBM is broadly described as measurement or quantification of biomarkers in human biological media such as blood or urine. Regarded as a widely recognized tool which is used to assess and evaluate exposure of general population, population groups, as well as individuals to environmental pollutants, several countries worldwide have successfully conducted large-scale studies and established reference values for metals and metalloids in diverse population. Perhaps, the largest, oldest and long-standing surveillance programme is the National Health and Nutrition Examination Survey (NHANES) in the USA initiated way back in 1971, followed by German Environmental Survey (GerES) in 1985, BIOAMBIENT. ES in Spain (2007) and France (2008) as well as Korea National Survey for Environmental Pollutants in the Human Body (KorSEP) in 2005 (Choi et al. 2017).

While countries from many parts of the globe have initiated human biomonitoring assessment, Malaysia falls short on this as we are yet to establish reference levels of populations exposed to diverse types of chemicals in the environment. To the best of our knowledge, no studies have been conducted thus far assessing pollutants in biological media on a nationwide scale among general population. However, studies pertaining to occupational exposure have been conducted in Malaysia including assessment of heavy metals in toenails of welders (Hariri et al. 2018; Zainal Bakri et al. 2019), cadmium exposure among waste collectors (Ismail et al. 2018), as well as heavy metals levels in nails of farmers (Ahmad Rohi et al. 2012).

Other related HBM studies from other parts of the world such as the European countries managed to identify subjects with an increased level of exposure (as opposed to background exposure) to a given environmental toxin. Using the baseline value of the general population may help toxicologists and physicians identify groups of individuals with higher body burden of the chemicals, thus enabling investigation and control measures to be carried out. In Malaysia, the scarcity of exposure assessment information prompted us to develop this study with the intention to obtain preliminary data on urinary levels of selected metals and metalloids among Malaysian adult population.

## Materials and Methods

### Study Design

This study is a collaboration between Environmental Health Research Centre (EHRC), Institute for Medical Research and Institute for Public Health. Urine samples were obtained from approved project entitled “Population-based Salt Intake Survey to Support the National Salt Reduction Programme for Malaysia (Malaysian Community Salt Study-MyCoSS)”. Since this study is an extension of the MyCoSS study, study design, sample size, sample collection, study instrument and other related parameters adhered to the MyCoSS study (Ambak et al. 2021).

MyCoSS was a cross-sectional population-based household survey. The inclusion criteria for the study were Malaysians aged more than 18 years old, residing in non-institutional living residences and agreed to participate in the study. Exclusion criteria include pregnant mothers, patients recently began diuretic therapy (< 4 weeks), having menses during urine collection, those diagnosed to have chronic diseases (such as kidney disease, heart failure or liver disease) and those having difficulty in collecting urine. To represent the Malaysian population, this survey conducted a stratified cluster sampling method. Sampling design covered both urban and rural areas for every state. Living quarters were randomly selected by the Department of Statistics Malaysia (DOSM). Only one participant was selected from a living quarter. If there were more than one eligible participant, selection was made using a modified Kish Table (Ambak et al. 2021).

This study was conducted between October 2017 and March 2018. Informed consent was obtained from all study participants prior to study commencement. For each subject, the information on age, gender, occupation, education background, health status, income, smoking status, marital status and other relevant information were obtained through a questionnaire which has been pre-tested and validated beforehand. Ethical approval of this study was received from the Medical Research and Ethics Committee [KKM.NIHSEC.P18-795(6)].

### Urine Collection and Analysis

Twenty-four-hour urine samples were collected from participants in clean, metal-free polyethylene containers (without preservatives or stabilizers). The final volume of urine collected for each respondent was recorded at the sampling site by the research team members. The decision to exclude urine samples less than 500 ml for laboratory analysis which were discarded at initial stage was decided by the parent study which this study diligently adheres to. Collected samples were transported to a private laboratory at 4 °C. Urine samples were homogenized and then aliquoted for heavy metal analysis and measurement of urine creatinine levels. Aliquoted urine in 15 ml polyethylene containers were sent to our laboratory and kept at − 80 °C until analysis for metals and metalloids.

Prior to analysis, urine samples were brought to room temperature, thawed, homogenized and diluted 1:10 in the same diluent as the corresponding calibration standards with 2% nitric acid (suprapur 65%). Lypochek Urine Metal Control for trace elements Level I and II (Bio-rad) was used for internal quality control. Recoveries for Ni, As, Cd, and Pb were between 85–115%. Observed values fell within the range of certified values. Metals and metalloid determinations were performed using ELAN 9000 ICP-MS (Perkin Elmer) with germanium (Ge), yitrium (Y) and terbium (Tb) used as internal standards. The calibration curves (5 points) were prepared within the concentration ranges normally observed in the general population from overseas studies (Pino et al. 2012; Aprea et al. 2018). The blanks for the analysis were prepared with 2% nitric acid. Limits of quantification (LOQ) of the blank measures were 0.27 µg/L for As, 0.05 µg/L for Cd, 1.40 µg/L for Ni, and 0.60 µg/L for Pb.

### Statistical Analysis

All analyses were performed using IBM SPSS 25. Urinary metal levels were expressed with respect to µg/g creatinine and log-transformed before analysis. Results with creatinine values below 0.3 and above 3.0 (too low or too high) (WHO 1996) were discarded from analysis. Data were tested for normality prior to analysis using Kolmogorov–Smirnov and Shapiro–Wilk tests. As the data were not normally distributed (*p* < 0.05), results were presented as geometric mean (GM) with 95% confidence interval. Statistical significance was established at *p* < 0.05. Determination of difference between two or more groups was analysed using Kruskal–Wallis test. Limit of detection (LOD) and limit of quantification (LOQ) were calculated as being, respectively, three and ten times the standard deviations of the concentrations of the blank samples. Data below LOD were replaced by value equal to half of the LOQ.

## Results

### Characteristics of Study Population

A total of 1440 adults participated in the study. Of the original sample, 429 individuals were excluded due to refusal of responding to questionnaire (*n* = 138), not a Malaysian resident (*n* = 35), sick (*n* = 16) and residence were locked during survey (*n* = 240). Another 194 individuals were excluded for reasons such as urine samples of less than 500 ml (*n* = 137) (Land et al. 2014), urine creatinine levels of < 0.3 g/L or > 3.0 g/L (WHO 1996)(*n* = 37), and cracked sample tubes (*n *= 20), leaving 817 individuals for present analysis. Although a total of 623 individuals were excluded from analysis, the findings did not differ from those included in the study.

The socio-demographics of respondents in this study was described and categorized by gender, age group, ethnic, education level, occupation, residence, smoking habit and Body Mass Index (BMI) as shown in Table 1. The number of male respondents was 346 (42.4%), while the female respondents were 471 (57.6%). Majority of respondents were above 45 years of age for both males: 64 (7.8%) and females 110 (13.5%). Most of the study population was Malay (64%), followed by Chinese (10.5%) and Indian (5.9%) which is coherent to ethnic distribution in Malaysia (DOSM 2020), predominantly being housewives (28.3%), possessed secondary education (61.8%), chiefly resided in rural (58%) as opposed to urban (42%), non-smokers (67.6%) and had an average BMI of 26.7 kg/m^2^ (± 5.3).

**Table 1:** Demographic characteristics of Malaysian by total population and gender

	Total N (%)	Men N (%)	Women N (%)
All	817 (100)	346 (42.4)	471 (57.6)
Age group (years)			
Mean ± S.D	48.9 ± 15.2	49.6 (15.7)	48.4(14.8)
18–24	55 (6.7)	24 (2.9)	31 (3.8)
25–34	123 (15.1)	53 (6.5)	70 (8.6)
35–44	134 (16.4)	49 (6.0)	85 (10.4)
45–54	174 (21.3)	64 (7.8)	110 (13.5)
55–64	193 (23.6)	91 (11.1)	102 (12.5)
> 65	138 (16.9)	65 (8.0)	73 (8.9)
Ethnic			
Malay	522 (64.0)	224 (27.4)	298 (36.5)
Chinese	86 (10.5)	33 (4.0)	53 (6.5)
Indian	48 (5.9)	13 (1.6)	35 (4.3)
Bumiputera Sabah/Sarawak	150 (18.4)	69 (8.4)	81 (9.9)
Others	10 (1.2)	6 (0.7)	4 (0.5)
Education level			
Primary	173 (21.2)	76 (9.3)	97 (11.9)
Secondary	505 (61.8)	211 (25.8)	294 (36.0)
Tertiary	79 (9.7)	42 (5.1)	37 (4.5)
None	60 (7.3)	17 (2.1)	43 (5.3)
Occupation			
Public sector	120 (14.7)	65 (8.0)	55 (6.7)
Private sector	126 (15.4)	71 (8.7)_	55 (6.7)
Housewives	231 (28.3)	0 (0)	231 (28.3)
Self-employed	180 (22.0)	111 (13.6)	69 (8.4)
Student	14 (1.7)	8 (1.0)	6 (0.7)
Unemployed	111 (13.6)	64 (7.8)	47 (5.8)
Others	35 (4.3)	27 (3.3)	8 (1)
Residence	343 (42.0)	146 (17.9)	197 (24.1)
Urban	474 (58.0)	200 (24.5)	274 (33.5)
Rural			
Smoking habit	552 (67.6)	108 (13.2)	444 (54.3)
Non-smoker	265 (32.4)	238 (29.1)	27 (3.3)
Smoker	160 (19.6)	151 (18.5)	9 (1.1)
Former smoker			
Body Mass Index (BMI-kg/m^2^)			
Mean ± S.D	26.7 ± 5.3	25.9 ± 4.8	27.3 ± 5.7
< 18.5 (Underweight)	35 (4.3)	17 (4.9)	18 (3.9)
18.5–24.9 (Normal weight)	288 (35.3)	132 (38.1)	156 (33.1)
24.9–29.9 (Overweight)	298 (36.5)	135 (39.0)	163 (34.6)
> 30 (Obese)	196 (23.9)	62 (18)	134 (28.4)

### Distribution of Metals and Metalloid in Urine

**Fig. 1:**
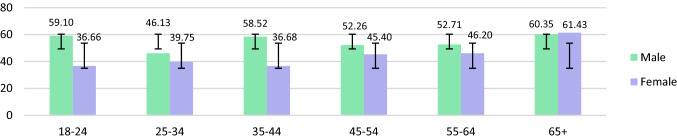
Urinary As concentrations (µg/L) between males and females of different age categories (Note: error bars represent standard deviation)

**Fig. 2:**
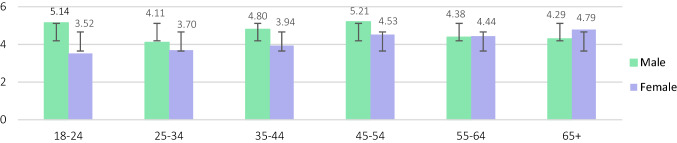
Urinary Ni concentrations (µg/L) between males and females of different age categories

**Fig. 3:**
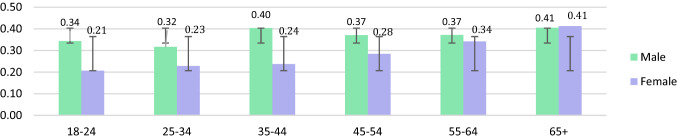
Urinary Cd concentrations (µg/L) between males and females of different age categories

**Fig. 4:**
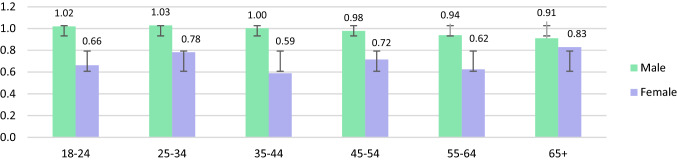
Urinary Pb concentrations (µg/L) between males and females of different age categories

Table 2 and 3 present the urinary metals results in µg/L and µg/g creatinine. The geometric mean of urinary metals concentrations among adults in Malaysia was As (48.21 µg/L; 95% CI: 44.82–51.83), Ni (4.37 µg/L; 95% CI: 4.15–4.61), Cd (0.32 µg/L; 95% CI: 0.30–0.34) and Pb (0.80 µg/L; 95% CI: 0.74–0.86). Urinary geometric mean was significantly higher in males as opposed to females for As (54.11 µg/L; 95% CI: 48.28–60.65, *p* < 0.003), Cd (0.37 µg/L; 95% CI: 0.34–0.41, *p* < 0.000) and Pb (0.97 µg/L; 95% CI: 0.86–1.09, *p* < 0.000). Smoking respondents showed significantly elevated urinary levels for Ni (4.73 µg/L; 95% CI: 4.29–5.21, *p* < 0.025), Cd (0.36 µg/L; 95% CI: 0.32–0.41, *p* < 0.007) and Pb (1.02 µg/L; 95% CI: 0.90–1.17 *p* < 0.000), but not for As. Those who reside in rural areas exhibited significantly higher As (51.60 µg/L; 95% CI: 46.93–56.75, *p* < 0.015), Cd (0.34 µg/L; 95% CI: 0.27–0.33, *p* < 0.044), and Pb (0.34 µg/L; 95% CI: 0.31–0.37, *p* < 0.036) urinary concentrations than those who reside in urban areas. The urinary concentrations were significantly different among respondents from various age groups only for Cd (*p* < 0.002), with respondents above 65 years of age showing the highest levels in females compared to males for As and Cd (Figs. 1, 2, 3, 4).

**Table 2:** Urinary metal concentrations levels (μg/L) in Malaysia by total population, gender, smoking status and strata

	N	AM	Sd	GM	95% CI (lower limits, upper limits)	Percentile				
						25th	50th	75th	90th	95th
Arsenic (As)										
Total	817	82.33	105.03	48.21	44.82, 51.83	24.14	47.76	103.06	194.81	259.48
Male	346	90.42	98.06	54.11	48.28, 60.65	27.62	55.06	124.05	221.26	283.20
Female	471	76.38	109.58	44.28	40.32, 48.63	22.69	43.54	92.30	162.37	238.23
Non-smoker	552	79.18	108.78	45.90	42.03, 50.14	23.80	45.58	94.58	175.21	252.92
Smoker	265	88.87	96.60	53.37	46.95, 60.65	26.70	54.29	123.50	221.56	271.09
Urban	343	77.51	115.00	43.86	39.19, 49.09	23.78	39.36	98.97	182.68	244.23
Rural	474	85.81	97.14	51.60	46.93, 56.75	25.81	54.11	104.62	212.13	280.03
Nickel (Ni)										
Total	817	5.73	4.34	4.37	4.15, 4.61	2.77	4.49	7.51	11.51	13.93
Male	346	6.17	4.86	4.57	4.19, 4.99	2.76	4.78	8.17	12.25	14.94
Female	471	5.41	3.89	4.24	3.96, 4.53	2.78	4.16	7.01	10.97	13.40
Non-smoker	552	5.46	4.03	4.21	3.96, 4.49	2.71	4.26	7.07	11.15	13.31
Smoker	265	6.29	4.89	4.73	4.29, 5.21	2.87	4.92	8.04	12.26	15.13
Urban	343	5.56	4.38	4.11	3.78, 4.49	2.58	4.30	7.35	11.53	14.13
Rural	474	5.85	4.31	4.57	4.28, 4.88	2.92	4.61	7.71	11.52	13.89
Cadmium (Cd)										
Total	817	0.47	0.43	0.32	0.30, 0.34	0.19	0.35	0.62	1.04	1.34
Male	346	0.54	0.49	0.37	0.34, 0.41	0.22	0.42	0.67	1.14	1.55
Female	471	0.43	0.37	0.29	0.26, 0.31	0.17	0.31	0.59	0.96	1.21
Non-smoker	552	0.44	0.38	0.30	0.28, 0.33	0.17	0.32	0.59	0.96	1.22
Smoker	265	0.54	0.51	0.36	0.32, 0.41	0.21	0.38	0.70	1.15	1.58
Urban	343	0.43	0.36	0.30	0.27, 0.33	0.18	0.32	0.59	0.90	1.16
Rural	474	0.51	0.47	0.34	0.31, 0.37	0.19	0.37	0.64	1.16	1.43
Lead (Pb)										
Total	817	1.53	2.21	0.80	0.74, 0.86	0.28	0.70	1.89	3.93	5.75
Male	346	1.75	2.13	0.97	0.86, 1.09	0.28	0.96	2.30	4.17	6.06
Female	471	1.38	2.26	0.69	0.63, 0.76	0.28	0.28	1.43	3.51	5.45
Non-smoker	552	1.38	2.16	0.71	0.65, 0.77	0.28	0.58	1.55	3.50	5.39
Smoker	265	1.86	2.29	1.02	0.90, 1.17	0.28	1.11	2.45	4.17	6.33
Urban	343	1.71	2.39	0.30	0.27, 0.33	0.28	0.80	2.31	4.32	6.20
Rural	474	1.45	2.07	0.34	0.31, 0.37	0.28	0.66	1.64	3.37	5.35

*AM* arithmetic mean, *Sd* standard deviation, *GM* geometric mean, *GSD* geometric standard deviation, *95% CI* 95% confidence interval

**Table 3:** Creatinine-corrected metal concentrations (μg/g) in Malaysian population

	N	AM	S.D	GM	95% CI (lower limits, upper limits)	Percentile				
						25th	50th	75th	90th	95th
Arsenic (As)										
Total	817	93.63	145.29	52.67	48.94, 56.69	25.94	51.00	105.85	207.78	313.91
Male	346	78.33	92.76	45.62	40.67, 51.18	22.92	46.02	95.87	178.44	271.27
Female	471	104.87	173.30	58.55	53.27, 64.36	28.66	57.16	109.19	235.99	325.69
Non-smoker	552	100.05	163.47	55.20	50.41, 60.44	27.03	54.94	107.52	234.64	316.45
Smoker	265	80.25	95.93	47.80	42.19, 54.14	24.34	44.57	100.53	185.82	277.65
Urban	343	85.32	144.31	46.91	41.95, 52.47	22.85	44.57	91.14	176.20	297.65
Rural	474	99.64	145.84	57.29	52.00, 63.12	27.90	60.93	115.98	225.22	317.10
Nickel (Ni)										
Total	817	6.41	5.04	4.78	4.52, 5.05	3.05	5.15	8.22	13.24	17.16
Male	346	5.26	4.31	3.85	3.53, 4.21	2.29	4.26	6.63	10.33	13.42
Female	471	7.26	5.36	5.60	5.22, 6.00	3.61	5.72	9.57	14.46	18.26
Non-smoker	552	6.76	5.06	5.06	4.73, 5.42	3.21	5.40	8.80	14.14	17.76
Smoker	265	5.68	4.91	4.23	3.84, 4.66	2.88	4.64	7.00	10.81	14.41
Urban	343	6.12	4.89	4.40	4.00, 4.83	2.85	5.02	8.03	13.12	17.48
Rural	474	6.62	5.13	5.07	4.73, 5.43	3.20	5.30	8.25	13.56	16.68
Cadmium (Cd)										
Total	817	0.53	0.50	0.35	0.33, 0.37	0.20	0.38	0.68	1.20	1.60
Male	346	0.47	0.48	0.31	0.28, 0.35	0.18	0.34	0.58	0.98	1.48
Female	471	0.57	0.52	0.38	0.35, 0.42	0.22	0.40	0.79	1.29	1.65
Non-smoker	552	0.55	0.49	0.36	0.33,0.40	0.20	0.39	0.77	1.26	1.64
Smoker	265	0.49	0.52	0.32	0.29, 0.36	0.18	0.36	0.59	0.99	1.52
Urban	343	0.47	0.44	0.32	0.29, 0.35	0.19	0.34	0.59	1.07	1.55
Rural	474	0.57	0.54	0.38	0.35, 0.41	0.20	0.41	0.79	1.25	1.65
Lead (Pb)										
Total	817	1.70	2.79	0.87	0.81, 0.94	0.35	0.76	1.88	4.29	6.28
Male	346	1.53	2.01	0.82	0.72, 0.92	0.26	0.85	1.88	3.63	5.36
Female	471	1.83	3.25	0.92	0.83, 1.01	0.37	0.71	1.87	4.74	6.54
Non-smoker	552	1.70	2.99	0.85	0.78, 0.93	0.35	0.67	1.82	4.42	6.31
Smoker	265	1.71	2.33	0.92	0.80, 1.05	0.31	0.98	2.08	3.86	6.19
Urban	343	1.83	3.19	0.94	0.84, 1.10	0.39	0.85	2.07	4.82	6.09
Rural	474	1.61	2.46	0.83	0.75, 0.91	0.33	0.71	1.85	3.91	6.35

*AM* arithmetic mean, *Sd *standard deviation, *GM* geometric mean, *GSD* geometric standard deviation, *95% CI* 95% confidence interval

### Comparison of Urinary Metals Concentrations Between Countries

The 95th percentile values for metals from this study were compared with values observed from international studies, namely, Germany, France, Flanders, USA, Canada, and South Korea as shown in Table 4. Results from this study showed that the 95th percentile values for urinary arsenic in Malaysian adult populations was found to be the highest between 2.2 times higher than adults in South Korea (Lee et al. 2012) to 17 times higher than adults in Germany (Wilhelm et al. 2004). Urinary Ni 95th percentile values in Malaysian adults were about 3 times higher than those in Belgium and Canada. On the other hand, urinary Cd 95th percentile value in Malaysia were comparable with Canadian adult population and seem to be in the intermediate range between the lowest urinary Cd concentrations in Flanders and the highest urinary Cd concentrations in South Korea.

**Table 4:** Comparison of urinary metals concentrations between countries

Country	Metals	95th percentile	Study population	Study period	Reference
Germany Belgium	As	15.0 µg/L 48.8 µg/L	Adults (age 18–69) Adults (age ≥ 18)	1997–1999 2010–2011	Wilhelm et al. (2004) Hoet et al. (2013)
Flanders		90.0 µg/L	Adults (age 20–40)	2007–2011	Schoeters et al. (2012)
USA		52.4 µg/L	Males and females (age ≥ 6)	2011–2012	CDC (2009)
Canada		76.0 µg/L	Canadian population (age 3–79)	2009–2011	CHMS (2013)
South Korea		119.7 µg/L	Adults (age ≥ 20)	2008	Lee et al. (2012)
Malaysia		260.0 µg/L	Adults (age 18–88)	2017–2018	This study
Belgium	Ni	4.73 µg/L	Adults (age ≥ 18)	2010–2011	Hoet et al. (2013)
Canada		4.80 µg/L	Canadian population (age 3–79)	2009–2011	CHMS (2013)
Malaysia		15.0 µg/L	Adults (age 18–88)	2017–2018	This study
Flanders	Cd	0.44 µg/L	Non-smoking adults (age 18–69)	1997–1999	Schoeters et al. (2012)
USA		0.87 µg/L	Males and females (age ≥ 6)	2011–2012	CDC (2009)
Canada		1.80 µg/L	Canadian population (age 3–79)	2009–2011	CHMS (2013)
South Korea		3.11 µg/L	Adults (age ≥ 20)	2008	Lee et al. (2012)
Malaysia		1.50 µg/L	Adults (age 18–88)	2017–2018	This study
Canada	Pb	1.90 µg/L	Canadian population (age 3–79)	2009–2011	CHMS (2013)
Malaysia		6.00 µg/L	Adults (age 18–88)	2017–2018	This study

## Discussion

This study aimed to determine the urinary As, Ni, Cd, and Pb levels among adults in Malaysia. To the best of our knowledge, this is the first study which successfully quantified metal levels in urine of Malaysian respondents on a national scale. This baseline information will contribute to identify Malaysian adults with high exposure group between 18 and 88 years of age. The discrepancies in values reported in other parts of the world might be explained by diverse environmental exposure profiles as well as unique dietary patterns of a particular nation. This is apparent in several studies conducted worldwide, as the values in this study are similar to those reported in certain areas, but could also be lower or higher depending on the vicinity of the study location.

It was reported that per capita fish consumptions by Malaysian adults was 54.77 kg/person/year in the year 2016, making it the second highest among Asian nations, after Japan (Nurul et al. 2016). Discrepancies between average per capita fish consumption exist and are considerably different across and within countries and regions due to the influence of culture, economy and geographical factors (FAO 2018). The fact that Malaysians are high fish consumers’ with the Malays significantly consuming fish higher than Chinese and Indians (Nurul et al. 2016) may well respond to the relatively high urinary total arsenic concentrations at 95th percentile (259.48 µg/L). Seafood intake markedly increase urine concentrations of total As as well as other As species (Choi et al. 2010; Navas-Acien et al. 2011) and respondents eating fish within 3 days showed significantly higher urinary As levels than those who did not (Lee et al. 2012). Other researchers also have established associations between urinary As excretion and consumption of seafood (Becker et al. 2003; Aguilera et al. 2008).

It is worth to note that toxicity of As largely depends on its oxidation states thus arsenic toxicity may be inaccurately assessed when analyses are restricted to total As alone. Speciation analysis is essential to distinguish the toxic and non-toxic fractions of the arsenic in specific media. Studies have shown that the proportion of arsenobetaine (organic As) in fish may be between 50% (Cubadda et al. 2017) and 87% (Lin et al. 2008) hence toxicity of dietary As exposure may have been overestimated if As in fish is presumed to be present exclusively as inorganic As. Inorganic As; the highly toxic form of arsenics are metabolized in the body as either monomethylarsonate (MMA) or dimethylarsonate (DMA)(Aposhian & Aposhian 2006). The ability of the body to metabolize either to MMA or DMA is profound to human health as MMA is linked to cause cancers and cardiovascular problems (Steinmaus et al. 2006; Wu et al. 2006).

In this study, the creatinine-adjusted urinary levels of As, Ni, Cd, and Pb were significantly higher in females than males. The patterns of occurrence by gender is similar to those reported in Italy for Ni (Aprea et al. 2018), Spain for Cd (López-Herranz et al. 2015) as well as in Korea for As (Park et al. 2016) by which the results might be influenced by the values of urinary creatinine. Due to higher muscle mass in males (Barr et al. 2005), creatinine concentrations are relatively higher for males than for females (Cocker et al. 2011). Differences in diet, behaviour and metabolism may explain the effects of gender on urinary metals levels (Arbuckle 2006) and the sex-related differences may also be influenced by physiological factors such as menstrual and reproductive factors (Lee and Kim 2014).

This study observed urinary As, Ni, Cd, and Pb levels which were higher in smokers rather than non-smokers. Studies by other researchers (Mortada et al. 2002; López-Herranz et al. 2015) revealed that subjects who were smokers had higher urinary Cd levels compared to subjects who did not smoke; thereby suggesting tobacco as main source of exposure in the general population (Jarup and Akesson 2009). Former and current smokers usually exhibit higher urinary cadmium levels as opposed to never-smokers (Olsson et al. 2002).

Taking age into account, urinary As, Cd, and Pb were significantly higher in males as opposed to females (*p* < 0.05) although no evident trend was observed between the males’ age categories. Females however showed upward trend for As, Ni, and Cd among those from different age categories while female respondents above 65 years old had the highest urinary As and Cd concentrations. Aprea et al. (2018) exhibited similar trends for urinary Cd levels in subjects above 40 years old as opposed to younger subjects. Some metals such as Cd and Pb may accumulate with age which may explain the higher urinary metals levels in the elder participants compared to the younger ones (Yasuda et al. 2012).

As biomonitoring studies in South East Asia is very scarce, established reference ranges from European and Asian population were used for comparison. Although these comparisons are quite biased as differences exist in terms of environmental exposures, geographical locations, eating habits, lifestyles and others, they do provide a benchmark for comparison among the observed elements of interest. Reference values as defined by the Commission on Human Biological Monitoring of the German Federal Environmental Agency is “intended to characterize the upper margin of the current background exposure of the general population to a given environmental toxin at a given time”. Reference values are strictly statistically derived values which is used to establish guideline values for population with increased internal exposure to environmental toxin and cannot be used to evaluate health-related criteria (Ewers et al. 1999).

On the other hand, human biomonitoring values (HBM) originated from toxicological and epidemiological studies hence can be used to represent health-related biological exposure limits by which two levels (HBM I & HBM II) were recommended by the commission. HBM I is the concentration of environmental toxin in biological media which pose no risk for adverse health effects to individuals in the general population (Ewers et al. 1999). HBM II is the concentration of environmental toxin in biological media which pose increased risk for adverse health effects in susceptible individuals of the general population (Ewers et al. 1999).

The health-based value for Cd in urine established by the German Human Biomonitoring Commission is 1 µg/L by which there is no risk for adverse health effects (HBM I)(Schulz et al. 2011). In this study, 10.5% of participants had Cd levels exceeding the HBM I levels suggesting that this population might be at risk as Cd is a recognized human carcinogen (IARC, category 1) and a well-known nephrotoxic agent. Low exposure to urinary Cd levels in the general population have shown substantial transformation in biomarkers for early kidney damage (Akesson et al. 2005; Noonan et al. 2002). Similar to As and Ni, urinary 95th percentile of Pb RV_95_ is about 3 times higher than that of the Canadians.

The 95th percentile for Malaysia was the highest for urinary As, Ni, and Pb except for Cd. Since this is the first biomonitoring study which is conducted in Malaysia, it is expected that the findings will be on the higher end of the spectrum. HBM data from other countries in Europe and Asia have seen reduction in levels of pollutants since the first HBM was initiated. This is possible as the available biomonitoring data was acted upon and measures are implemented to reduce exposure among the general population after series of HBM surveys. For instance, the US Environmental Protection Agency (EPA) has set the maximum arsenic level in public water systems at 50 µg/L for decades and in January 2006, the level was set to 10 µg/L. A study by Nigra et al. (2003) showed that the implementation of the new regulation has seen reductions in drinking water arsenic exposure among public well water users thereby confirming vital role of federal drinking water regulations in reducing toxic exposures and protecting human health. On the other hand, the findings from this study will provide stakeholders and policy makers with urinary metals and metalloids data hence appropriate risk reduction strategies could be executed.

This research has some limitations. First, the ratios of age and gender were greatly skewed in this study. The study population was mostly females and majority of the respondents were more than 45 years old. The data collection more often than not was conducted during working hours therefore mostly housewives were available as well as the elderly. Second, this study measures only total As concentrations in urine instead of As speciation. Thus, interpretation of As in urine may be less accurate and overrated as fraction of organic As (non-toxic) is usually more than inorganic As (toxic). Third, details on fish consumption from the respondents were not available hence only assumptions could be made that the respondents with high urinary As concentrations may have eaten fish or seafood prior to 24 h urine sampling. Fourth, this study measures urine; which reflects current exposure rather than hair or nails which could reflect long-term exposure in humans. Fifth, collection of 24 h urine samples was a cumbersome process as it critically affects response rate. Volume of urine sample less than 500 ml was decided to be discarded by the parent study therefore no urine samples were received for that particular individuals. As aforementioned, samples included for analysis continues to be representative of the original study sample. Hence, future study should consider collection of spot urine samples instead of 24 h urine samples. Lastly, comparison between findings from this study and other studies in Malaysia is very challenging as other studies measured either urine in occupationally exposed population or trace elements concentrations in hair.

## Conclusions

In conclusion, the urinary concentrations of arsenic, nickel, cadmium and lead obtained from this study provided information on urine metals and metalloid levels in Malaysian adults. This study measured population body burden in adults and evaluated effects of gender, age and smoking status influencing urinary exposure. It should be noted, however, that this study did not conduct arsenic speciation; hence future studies should include measurement of inorganic and organic species to differentiate the proportion of toxic and non-toxic fraction which are mainly derived from fish and seafood consumption. In addition, future studies should also include respondents’ information on fish and seafood consumption or exclude those who eat fish and seafood entirely from being study subject. Subjects younger than 18 years old should be included as study subjects to enable a more comprehensive biomonitoring data.
